# The Effect of Gravity on Marginal Integrity of Different Flowable Bulk-Fill Resin Composites

**DOI:** 10.3390/medicina60030396

**Published:** 2024-02-26

**Authors:** İsmail Hakkı Baltacıoğlu, Gülbike Demirel, Mehmet Eray Kolsuz, Kaan Orhan

**Affiliations:** 1 Department of Restorative Dentistry, Faculty of Dentistry, Ankara University, 06560 Ankara, Turkey; baltacioglu@ankara.edu.tr; 2 Department of Dentomaxillofacial Radiology, Faculty of Dentistry, Ankara University, 06560 Ankara, Turkey; ekolsuz@ankara.edu.tr (M.E.K.); knorhan@dentistryt.ankara.edu.tr (K.O.); 3 Department of Oral Diagnostics, Faculty of Dentistry, Semmelweis University, 1085 Budapest, Hungary

**Keywords:** bulk-fill resin composites, gravity, marginal integrity, micro-ct

## Abstract

*Background and Objectives*: The aim of this quantitative research was to investigate the effect of gravitational forces on the marginal integrity of different bulk-fill composites by micro-CT imaging. *Materials and Methods*: Fifty caries-free human third molars extracted for prophylactic purposes were used in this study. Each tooth was prepared with two proximal box cavities, with dimensions of 3 mm × 3 mm × 5 mm. Five distinct groups, each comprising 20 cavities, thus totaling 100 cavities for this study: (1, Group CON): Clearfil Majesty Flow + Clearfil Majesty Esthetic (as the control); (2, Group FBR): Filtek Bulk-fill Flowable Restorative + Clearfil Majesty Esthetic; (3, Group XTB): Voco Extrabase + Clearfil Majesty Esthetic; (4, Group SDR): SDR + Clearfil Majesty Esthetic; and (5, Group SNC): Sonicfill. When restoring the mesial cavities, the occlusal surfaces of the teeth in the mold were positioned upwards, counteracting the force of gravity. In contrast, for the restoration of the distal cavities, the occlusal surfaces were aligned downwards, to be parallel with the gravitational pull. After restorative procedures, each tooth was treated with 5000 thermal cycles. A solution of ammoniacal silver nitrate (AgNO_3_) was employed as a tracing agent. The micro-CT scans were conducted and the total volume of silver nitrate and the total volume of restorations within the relevant region of interest were calculated in “mm^3^” with software. Two-way ANOVA and Tukey tests were performed at a significance level of *p* = 0.05 with Graphpad Prism v 8.2.1 software. *Results*: Both gravity effect and interaction showed no statistical differences (*p* > 0.05). Statistically significant differences were observed in the restorative materials (*p* < 0.05). *Conclusions*: Gravitational forces do not emerge as a major factor affecting the marginal integrity of flowable bulk-fill composites in class II restorations. The chemical composition of the composites plays a more crucial role, with the XTB composite showing higher microleakage ratios compared to the others.

## 1. Introduction

Resin composites, despite their widespread categorization, exhibit numerous variations based on characteristics such as viscosity, placement technique, application area, and shade [1,2,3,4]. These variations are reflected in their different usage scenarios. Dental resin composites are broadly classified based on their viscosity into two categories: flowable and paste-like consistencies [5]. Flowable composites also exhibit variations in composition and physicochemical properties across different manufacturers [6]. Traditional and bulk-fill composites differ significantly, especially in their increments of application. Bulk-fill composites can be applied in significantly thicker layers, up to 4–6 mm in some cases, and even as a single layer, offering both time efficiency and reduced precision requirements during placement [7,8]. There are two distinct types of bulk-fill composites depending on their viscosity—paste-like and flowable. Paste-like composites are typically used as a full body due to their high filler content and resistance to wear [9], while flowable composites often serve as base materials due to their lower filler content and reduced wear resistance [10,11].

As with all restorative materials, resin composites are expected to serve in the oral environment over the long term; however, deteriorations in marginal integrity can lead to the failure of these restorations and necessitate their replacement [12]. Ensuring marginal integrity is a critical aspect of restorative materials. The marginal integrity of dental composites is influenced by polymerization shrinkage, adhesive bonding effectiveness, curing techniques, differences in thermal expansion coefficients, moisture contamination, operator skill and technique, material composition, occlusal stress, quality of finishing and polishing, and material aging [13]. These factors can potentially lead to gaps, causing issues such as microleakage, sensitivity, and compromised restoration longevity [14]. Micro-computerized tomography (Micro-CT) has proven to be a reliable technique to investigate marginal integrity, allowing non-destructive three-dimensional analysis of desired sample regions.

The cavities prepared for restorative procedures in the oral environment have different spatial locations. These differences are due to the region of the tooth to be restored, the position of the patient’s head while sitting in the dental unit, the position that the operator sets for the patient’s head during the procedure, and whether the tooth is located in the lower or the upper jaw [15]. This spatial configuration leads to different gravitational forces acting on the cavity while the restorative material is being placed. In the literature, nearly all class II microleakage studies conducted to date have been performed in the classic manner, oriented in the direction of gravitational forces, and there is no study investigating the effects of such gravitational forces acting on the cavity that could lead to interfacial voids while placing the different flowable restorative materials and thus affecting the marginal integrity of flowable materials and no study has yet to investigate this so far. Therefore, this quantitative research study aims to analyze the effect of gravitational forces on the marginal integrity of different bulk-fill composites by Micro-CT imaging. The null hypotheses tested were (1) gravitational forces have no effect on marginal integrity, (2) different flowable bulk-fill composites show similar marginal integrity.

## 2. Materials and Methods

The research adhered to the ethical guidelines set forth by the Ankara University Faculty of Dentistry’s Committee for Human and Animal Studies (approval reference: 36290600/35). For statistical analysis, the present study employed the GPower 3.1 program (Düsseldorf, Germany), setting the significance level at α = 0.05 and the power at β = 0.80. The effect size was determined based on a review of similar studies in the existing literature [16,17], establishing a minimum of 10 samples for each group as adequate.

For this study, 50 caries-free human mandibular third molar teeth, extracted for prophylactic reasons, were used; adhering to predefined cavity dimensions to ensure uniformity and maximal similarity among the samples. The teeth were preserved in a saline solution maintained at +4 °C and were utilized within a week following extraction. Initial preparation involved the removal of any lingering tissue remains, followed by thorough cleaning with pumice and evaluation for cracks and fractures. Teeth with defects were excluded from the present study. For each tooth, two proximal box cavities were prepared, mesial and distal class II box cavities with similar dimensions (≈3 mm × 3 mm × 5 mm). The gingival floor of these cavities, at the cervical end, was set 1 mm below the cemento–enamel junction. The cavity formation process included the use of new cylindrical diamond burs (Hager & Meisinger GmbH, Neuss, Germany) and a high-speed handpiece with water cooling. The gingival margins of these cavities were prepared as straight edges, not beveled.

The depth of each cavity was standardized to 5 mm, achieved by evenly grinding the occlusal surfaces of the teeth under water cooling using carbide abrasive papers (grades P1000 to P4000, Metkon, Gripo 2v Grinder-Polisher, Bursa, Turkey). To ensure that the cavities were consistent in size across all samples, measurements were taken using digital calipers (Model 500 by the Mitutoyo Corporation, Kanagawa, Japan).

### 2.1. Restorative Procedures

**Figure 1:**
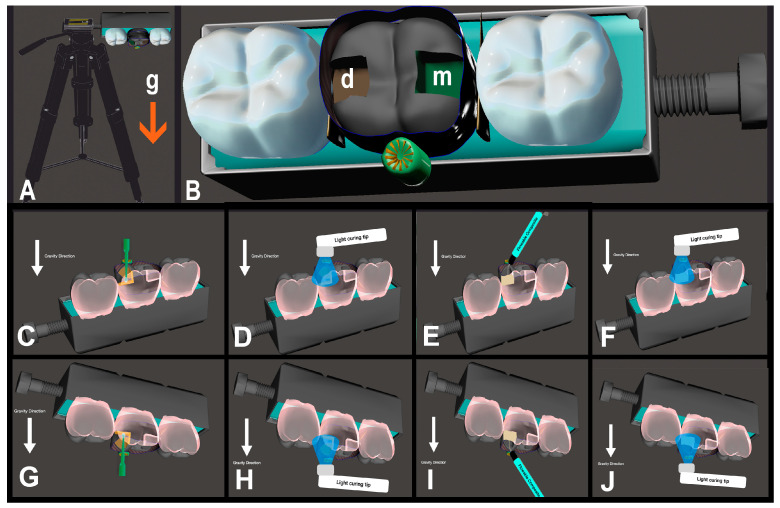
(**A**): Restoration template with tripod; (**B**): closer occlusal view of the template, g: direction of gravity force, m: mesial cavity box, d: distal cavity box; (**C**): adhesive application with gravity force; (**D**): light-curing of adhesive with gravity force; (**E**): restorative application with gravity force; (**F**): light-curing of restorative with gravity force; (**G**): adhesive application against gravity force; (**H**): light-curing of adhesive against gravity force; (**I**): restorative application against gravity force; (**J**): light-curing of restorative against gravity force. (The white arrow indicates the direction of gravity).

Once the preparatory steps were finalized, the teeth were randomly assorted into five distinct groups, each comprising 20 cavities, thus totaling 100 cavities for this study: (1, Group CON): Clearfil Majesty Flow + Clearfil Majesty Esthetic (as the control); (2, Group FBR): Filtek Bulk-fill Flowable Restorative + Clearfil Majesty Esthetic; (3, Group XTB): Voco Extrabase + Clearfil Majesty Esthetic; (4, Group SDR): SDR + Clearfil Majesty Esthetic; and (5, Group SNC): Sonicfill. Detailed information on these materials is provided in Table 1. The process of restoration was guided using a template depicted in Figure 1. Each acrylic tooth was set in self-curing acrylic resin, placed within putty silicone material along with other teeth. A screw mechanism was utilized to adjust and maintain the positioning of the teeth in contact with one another. The restoration template was secured on an adjustable tripod for stability. When restoring the mesial cavities, the occlusal surfaces of the teeth in the mold were positioned upwards, counteracting the force of gravity. In contrast, for the restoration of the distal cavities, the occlusal surfaces were aligned downwards, to be parallel with the gravitational pull.

During the restoration process, Adapt SuperCap Matrix bands (no 2182; Kerr-Hawe, Bioggio, Switzerland) were placed around the teeth, and each cavity was treated with a layer of Clearfil S^3^ Bond Plus bonding agent (Kuraray America Inc., New York, NY, USA), with agitating for 20 s; then, dried with a gentle steam of air for 5 s and light cured for 10 s with SDI Radii Plus light curing unit (SDI Limited, Bayswater, Australia), with the unit’s output power regularly checked with a radiometer to maintain a minimum intensity of 1000 mW/cm^2^. The application of the composites followed the group assignments as listed in Table 1. For the paste-like composites, hand instruments were used to apply the material under vertical pressure. In contrast, the flowable composites were introduced using their designated applicator tips and then contoured to the cavity walls using a dental probe. The technique of horizontal incremental layering was employed for all types of restorations. The curing of each layer of composite material was achieved using the same light curing unit. All cavities were prepared and restorative procedures were conducted by a single operator, a restorative specialist with 10 years of experience. In samples conducted against gravity, the operator performed the procedures in an upward position by reclining the dental unit.

After restorative procedures, each tooth treated with 5000 thermal cycles, alternating between 5 °C and 55 °C, with each cycle lasting 60 s, to simulate aging through thermal cycling. Following this, a solution of ammoniacal silver nitrate (AgNO_3_) was employed as a tracing agent to infiltrate microscopic gaps at the dental restoration–tooth interface. The procedure involved immersing the samples in a 50% AgNO_3_ solution for 12 h in a dark environment to prevent premature ion reduction, followed by a thorough rinse under tap water for five minutes. Subsequently, the teeth were submerged in a photo-developing solution and then exposed to light for a duration of eight hours in order to turn silver ions in penetrated areas into visible metallic silver marks to highlight marginal gaps for analysis [18]. Before proceeding with Micro-CT scanning, the teeth were meticulously cleaned using a toothbrush and then polished with aluminum oxide discs to remove any residual silver deposits.

### 2.2. Micro-CT Scanning

The Micro-CT scans were conducted using a Bruker Skyscan 1172 high-resolution desktop system (Kontich, Belgium). The scanning parameters set were 100 kilovoltage peak (kVp), 100 milliampere-seconds (mA), and a filter combination of 0.5 mm aluminum/copper (Al/Cu). The pixel sizes selected for scanning were 5.2, 8.1, 11.2, and 13.74 μm, with the specimens being rotated incrementally by 0.5 degrees. Air calibration of the detector was routinely performed before each scan to reduce the occurrence of ring artifacts. The total rotation for each sample was 360 degrees, with an integration time of 5 min per scan, averaging a total scanning duration of approximately 2 h. Additional settings included adjustments for beam hardening and setting the optimal contrast limits as per the manufacturer’s recommendations, tailored to the specific characteristics of the teeth based on previous scans and reconstructions.

### 2.3. Micro-CT Analysis

**Figure 2:**
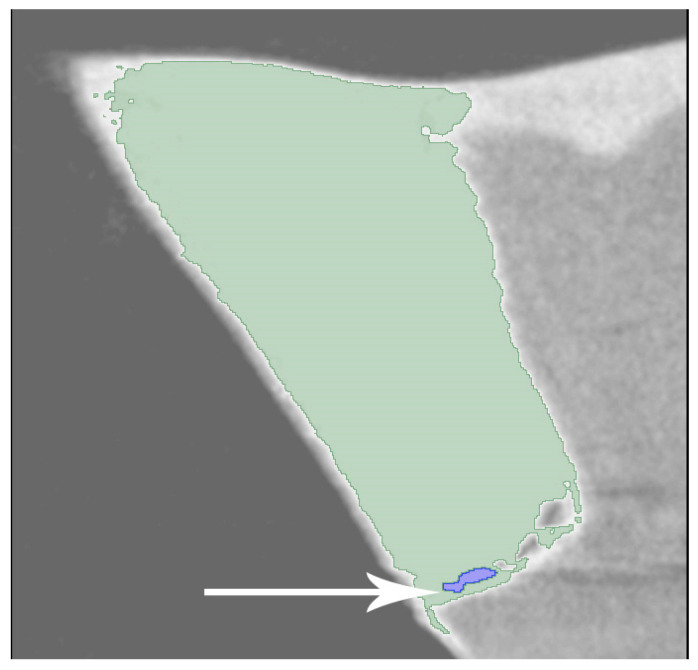
The white arrow indicates the silver nitrate on the toot-restoration interface.

Data reconstruction was carried out using the NRecon software (version 1.6.10.4; SkyScan, Billerica, MA, USA), which incorporates a modified algorithm originally developed by Feldkamp and colleagues. This software facilitated the generation of axial, two-dimensional images with a resolution of 1000 × 1000 pixels. During the reconstruction process, specific parameters were set, with ring artifact correction established at level 7 and smoothing at level 3, both maintained at zero, while the beam artifact correction was adjusted to 38%. The reconstructed images, produced using NRecon, displayed two-dimensional cross-sections of the dental crowns. The resulting images were analyzed with the Slicer 4.10.1 software. In the program, different threshold values of restorative material, silver nitrate, enamel, and dentin tissues were determined (Figure 2). The total volume of silver nitrate and the total volume of restorations within the relevant region of interest were calculated in “mm³”. These values were then divided to determine the leakage rates for the respective samples. The Graphpad Prism v 8.2.1 (San Diego, CA, USA) program was used for statistical analysis. Differences among restorative materials in terms of silver-nitrate leakage rates were normally distributed, thus, two-way ANOVA was performed, and the Tukey test was performed for multiple comparisons at a significance level of *p* = 0.05.

## 3. Results

**Figure 3:**
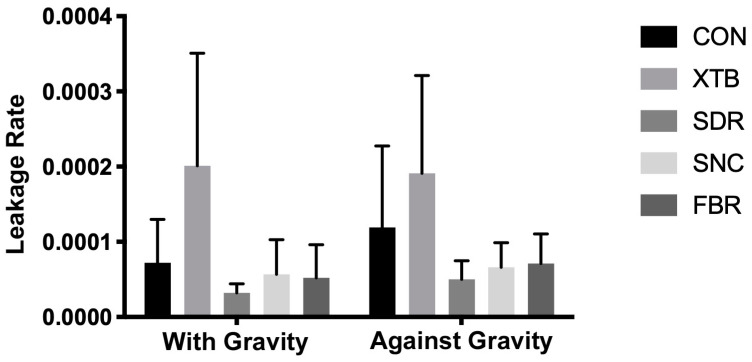
Microleakage rates.

The mean leakage percentage values are shown in Figure 3. According to the two-way analysis of variance (Table 2), statistically significant differences were observed in restorative materials (*p* < 0.0001). Both the gravity effect and interaction showed no statistical differences (*p* > 0.05).

When assessing the composite effect in groups restored through gravitational force (as the traditional methodology setup), a statistically notable increase in leakage rate was observed in the XTB group compared to all other tested groups (*p* < 0.05). There was no significant difference between the CON, SDR, SNC, and FBR groups (*p* > 0.05).

In groups restored against gravity force, a statistically significant higher leakage rate was shown for the XTB group than all other tested groups (*p* < 0.05), except the CON group (*p* > 0.05). There was no significant difference between CON, SDR, SNC, and FBR groups (*p* > 0.05).

## 4. Discussion

Restorative materials, which are used to replace decayed, lost, or removed tooth tissue, are expected not to exhibit microleakage and to demonstrate good marginal integrity [19]. The effects of the viscoelastic properties of flowable composite resins in the oral environment, under different head positions and across various tooth regions, on microleakage are not well understood. Considering these factors, the present study aimed to evaluate the marginal adaptation of bulk-fill materials in class II restorations, which are among the most used areas clinically, using Micro-CT and applying them under different gravitational forces. In this study, marginal adaptation was assessed using micro-computed tomography (Micro-CT) and three-dimensional (3D) measurement software. This non-destructive technique allowed for volumetric evaluations across all relevant surfaces of the samples without causing any harm [20,21]. The thermo-cycling process is essential in dental studies for mimicking the oral environment’s thermal changes, critically affecting the assessment of marginal integrity in dental materials and restorations [22]. To consider the effects of temperature variations in the oral environment, a thermal aging protocol was applied.

In this study, the incorporation of gravitational forces was motivated by the inquiry into whether materials with a flowable consistency, commonly employed in restorative procedures, particularly for maxillary dental restorations within the oral environment, may influence marginal integrity. The primary focus is directed towards ascertaining if the inherent fluidity of these materials contributes to their displacement from the tooth surface, consequently impacting the precision of marginal integrity in restorative applications. The findings of this study revealed that gravitational forces do not impact the marginal integrity of bulk-fill composites. Therefore, our first study hypothesis was accepted. As there are no previous studies examining gravity-related microleakage, there are no data points for comparison with the results of this study. Nevertheless, certain assumptions can be made regarding this situation. Despite the expectation of flow or movement in the direction of gravity in restorations involving the use of flowable composites against gravity, our study did not reveal any significant alteration in terms of marginal adaptation. However, further investigations are imperative to comprehensively assess the precise impact of this phenomenon on a restoration. Additional studies, employing similar or analogous experimental setups, focusing on bond strength assessments, investigations into polymerization vectors, and evaluations of the restoration–tooth interface with electron microscopes, could provide more insights into the influence of this concept. Additionally, there is a specific limitation associated with this methodology; the present study solely investigated the effects of force in two distinct directions. The significance of gravity in a clinical scenario remains controversial. There exist infinite permutations of tooth, cavity, location, operator’s position, patient’s position, and restorative technique, with the potential for these variables to undergo changes within the same procedure [23]. Consequently, this factor may be deemed insufficient in use with a two-direction method.

Contrary to our second null hypothesis, the XTB composite demonstrated a statistically significant higher leakage rate when compared to other bulk-fill groups in both “with gravity” and “against gravity” groups.

Numerous studies have been conducted on the microleakage and marginal integrity of bulk-fill composites [24,25,26]. While some of these studies have found no significant differences among various bulk-fills, others have identified variations. In parallel to this study, some research comparing XTB with other bulk-fill composites has found XTB to exhibit lower marginal integrity [27,28,29]. The reduced integrity of XTB may be attributed to its chemical composition. While many bulk-fill composites blend traditional and new monomers, XTB’s structure uniquely consists solely of BIS-EMA as its monomer and a higher filler level of (w: %76). This difference could potentially influence polymerization stresses, thereby affecting the marginal adaptation compatibility. The lack of comparative studies on the viscoelastic properties and behaviors during polymerization among the XTB, FBR, SNC, and SDR composites utilized in this study hinders a comprehensive explanation of the observed lower adaptation of XTB.

One of the implications drawn from this study is that three bulk-fill (SDR, SNC, FBR) composites exhibit superior marginal adaptation compared to the traditional flowable composite (CON), both in the direction of gravity and against gravity. XTB, while demonstrating better adaptation to gravitational forces, exhibits a similar marginal fit to traditional flowable composite (CON) in the direction of gravity. This suggests that bulk-fill composites may be recommended for use instead of traditional composites in clinical applications.

Addressing the complexities of selecting comparable teeth and the experimental nature of our study underscores the potential for variations in outcomes, highlighting the need for refined methodologies to ensure uniformity. Our investigation into the effects of gravitational forces on the marginal integrity of bulk-fill composites, while revealing no significant impact, opens avenues for further research into other influencing factors. Additional studies could also investigate the interplay between the chemical properties and the rheological behavior of these materials to develop a more comprehensive understanding that can inform both material formulation and clinical application techniques. Nonetheless, we acknowledge a few potential limitations of our study, including the unidirectional evaluation of gravitational force, the uncertain impact of the metal matrix band used on the viscoelastic properties of the restorative material, the distinctive application technique of SNC compared to other bulk-fill composites, and the use of a single type of adhesive system. These considerations emphasize the necessity for ongoing research in this domain to deepen our understanding of these materials’ behavior in diverse and dynamic oral environments. The most significant limitation in this study is undoubtedly the operator’s position during restorative procedures. The application of operative tasks from different angles can lead to varying outcomes, both clinically and in laboratory studies. Therefore, gravity alone is not the sole factor.

## 5. Conclusions

Although there is a gap in the literature regarding this issue, in conclusion, this research indicates that gravitational forces are not a major factor affecting the marginal integrity of flowable bulk-fill composites in box-only class II restorations. Rather, the marginal integrity appears to be more significantly influenced by the chemical composition of the composite. Notably, the x-tra base group exhibited higher microleakage ratios compared to other bulk-fill composites. All bulk-fill composites can be clinically utilized as alternatives to traditional ones.

## Figures and Tables

**Table 1:** Materials used in this study and their application protocols.

Group Abbreviation	Restorative Material(s)	Manufacturer	Composition	Application Method	Number of Layer(s)
-	Clearfil Majesty Esthetic (Paste Like Restorative material)	Kuraray Noritake, Japan	Bis-GMA, TEGDMA, Barium glass filler, silica filler. Filler Content (wt.%): 85.5	Used as “capping material” with incremental technique described in the groups below.	-
CON	Control: Clearfil Majesty Flow	Kuraray Noritake, Japan	Hydrophobic aromatic, dimethacrylate, TEGDMA, camphoroquinone, barium glass filler, silica filler. Filler Content (wt.%): 81	A 1 mm increment of a flowable composite was applied on the gingival floor. The thickness was confirmed using a periodontal probe, and light cured for 20 s. A 1 mm increment of the CME composite material was applied and light cured for 20 s. The rest of the cavity was restored using 1.5 mm increment of Clearfil Majesty Esthetic composite and light cured for 20 s.	4 (base, intermediate, top)
FBR	Filtek Bulk-fill Flowable Restorative	3M Dental Products. St. Paul, USA	Silane treated ceramics, UDMA, BIS-EMA-6, YbF_3_, BISGMA, TEGDMA, ethyl 4-dimethylaminobenzoate. Filler Content (wt.%): 65	A 4 mm increment of a bulk fill composite was applied on the gingival floor. The thickness was confirmed using a periodontal probe, and light cured for 20 s. The rest of the cavity was restored using 1 mm increment of Clearfil Majesty Esthetic composite and light cured for 20 s.	2 (base, top)
XTB	Voco Extra Base	VOCO GmbH, Germany	Bis-EMA, aluminum, and barium silicate. Filler Content (wt.%): 75	A 4 mm increment of a bulk fill composite was applied on the gingival floor. The thickness was confirmed using a periodontal probe, and light cured for 20 s. The rest of the cavity was restored using 1 mm increment of Clearfil Majesty Esthetic composite and light cured for 20 s.	2 (base, top)
SDR	Surefill SDR	Dentsply DeTrey, Konstanz, Germany	Modified UDMA, TEGDMA, EBPDMA, pigment, photoinitiator, barium and strontium alumino-fluoro-silicate glasses, silicon dioxide-amorphous, strontium. Aluminosilicate glass. Filler Content (wt.%): 68	A 4 mm increment of a bulk fill composite was applied on the gingival floor. The thickness was confirmed using a periodontal probe, and light cured for 20 s. The rest of the cavity was restored using 1 mm increment of Clearfil Majesty Esthetic composite and light cured for 20 s.	2 (base, top)
SNC	SonicFill II	Kerr, Orange, CA, USA	Bis-GMA, TEGDMA, Bis-EMA, silicon dioxide, glass, oxide, chemicals. Filler Content (wt.%): 83	A 5 mm increment of a bulk fill composite was applied on the whole cavity with Sonicfill device and light cured for 10 s from occlusal, 10 s from buccal, and 10 s from lingual wall.	1 (bulk)

Chemical Abbreviations: Bis-GMA: bisphenol-A glycidyl methacrylate; TEGDMA: triethylene glycol dimethacrylate; UDMA: urethane dimethacrylate; BIS-EMA: ethoxylated bisphenol-A dimethacrylate; YbF3: ytterbium (III) fluoride; EBPDMA: ethoxylated bisphenol-A dimethacrylate; Bis-EMA: bisphenol-A ethoxylate dimethacrylate.

**Table 2:** Results of the 2-way ANOVA and Tukey Tests (*: indicates significant difference).

ANOVA Table	SS	DF	MS	F (DFn, DFd)	*p* Value
Interaction	8 × 10^−9^	4	2 × 10^−9^	F (4, 90) = 0.3425	0.8486
Gravity Factor	7 × 10^−9^	1	7 × 10^−9^	F (1, 90) = 1.112	0.2944
Composite Factor	3 × 10^−7^	4	8 × 10^−8^	F (4, 90) = 12.34	* <0.0001
Residual	6 × 10^−7^	90	6 × 10^−9^		
			Tukey’s Multiple Comparison		
Groups		With Gravity		Against Gravity	
		Mean Diff.	*p* value	Mean Diff.	*p* value
CON vs. XTB		−13 × 10^−5^	* 0.0037	−7 × 10^−5^	0.2531
CON vs. SDR		4 × 10^−5^	0.7868	7 × 10^−5^	0.2938
CON vs. SNC		2 × 10^−5^	0.9930	5 × 10^−5^	0.5615
CON vs. FBR		2 × 10^−5^	0.9793	5 × 10^−5^	0.6522
XTB vs. SDR		17 × 10^−5^	* <0.0001	14 × 10^−5^	* 0.0012
XTB vs. SNC		14 × 10^−5^	* 0.0009	13 × 10^−5^	* 0.0054
XTB vs. FBR		15 × 10^−5^	* 0.0005	12 × 10^−5^	* 0.0085
SDR vs. SNC		−3 × 10^−5^	0.9537	−2 × 10^−5^	0.9911
SDR vs. FBR		−2 × 10^−5^	0.9793	−2 × 10^−5^	0.9753
SNC vs. FBR		0.5 × 10^−5^	>0.9999	−0.5 × 10^−5^	>0.9999

## Data Availability

Data available upon request.
